# CCR7^+^ selected gene-modified T cells maintain a central memory phenotype and display enhanced persistence in peripheral blood in vivo

**DOI:** 10.1186/s40425-017-0216-7

**Published:** 2017-02-21

**Authors:** Gray Kueberuwa, Hannah Gornall, Erik Marcelo Alcantar-Orozco, Deborah Bouvier, Zainul Abedin Kapacee, Robert Edward Hawkins, David Edward Gilham

**Affiliations:** Clinical and Experimental Immunotherapy Group, Manchester Cancer Research Centre, Faculty of Biology, Medicine and Health, Division of Cancer Sciences, The University of Manchester, Wilmslow Road, Withington, Manchester, M20 4QL UK

**Keywords:** CCR7, CD62L, Adoptive cell therapy, Immunotherapy, Differentiation status, Transgenic TCR, Central memory, DMF5

## Abstract

**Background:**

Adoptive T cell immunotherapy (ATCT) for cancer entails infusing patients with T cells that recognise and destroy tumour cells. Efficient engraftment of T cells and persistence in the circulation correlate with favourable clinical outcomes. T cells of early differentiation possess an increased capacity for proliferation and therefore persistence, using these cells for ATCT could therefore lead to improved clinical outcomes.

**Method:**

We describe a method to enrich T cells of early differentiation status using paramagnetic beads and antibodies targeting cells expressing C-C motif chemokine receptor 7 (CCR7).

**Results:**

Selection of cells expressing CCR7 enriches T cells of bearing markers of early differentiation status. This was validated through analysis of an array of surface markers and an observed reduction in effector cell functions ex vivo. CCR7 selection resulted in dramatic 83.6 and 137 fold increases in circulating levels of CD4 and CD8 T cells respectively compared to non-sorted T cells 3 weeks after adoptive transfer to NSG mice. We observed no significant difference in the engraftment levels of CCR7 or CD62L selected cells in the NSG mouse model. Comparison of cells ex vivo, however, suggests CCR7 selection is superior to CD62L selection in enriching T cells of early differentiation status.

**Conclusions:**

CCR7 selection offers a means to enrich T cells of early differentiation status for ACTC. Together our data suggests that these T cells are likely to display enhanced engraftment and persistence in patients in vivo and could therefore improve therapeutic efficacy of ACTC.

**Electronic supplementary material:**

The online version of this article (doi:10.1186/s40425-017-0216-7) contains supplementary material, which is available to authorized users.

## Background

Adoptive T cell therapy (ATCT) using T cells armed with recombinant T Cell Receptor (TCR) and Chimeric Antigen Receptor (CAR) technologies is showing highly encouraging activity in early phase clinical testing against several malignancies across a number of Institutions [1]. An emerging theme is that efficient engraftment and long-term persistence of the therapeutic T cells correlates with positive therapeutic outcomes. Several pre-clinical studies have shown that naïve and early-differentiated T cells possess an enhanced capacity for long term persistence [2–4] and can elicit potent anti-tumour responses [5–8]. Additionally, the increased persistence of adoptively transferred cells appears to be dependent upon the acquisition of central memory T cell (T_CM_) populations [9–12].

Stable gene transfer has been routinely achieved in the clinical setting through the use of gamma retroviral vectors to transduce polyclonal T cells with CARs [13–18] and TCRs [19–21] with these engineered cells showing no obvious adverse safety indications in patients engrafted with CAR T cells for greater than 10 years [22]. For efficient transduction with retroviral vectors, primary T cells need to be actively proliferating [23] which is generally achieved through the mitogenic stimulation of resting primary T cells by activation of the TCR/CD3 complex to deliver ‘signal 1’ and through co-stimulatory pathways such as CD28 to provide ‘signal 2’. Lentiviral vectors are now commonly used in place of gamma-retrovirus vectors for CAR and TCR gene expression although primary T cells still require mitogenic activation for efficient transduction with standard lentiviral vectors used for research and clinical work [24, 25].

Upon activation, T cells progress in an irreversible linear fashion towards an effector (T_E_) phenotype [26, 27]. Mitogenic activation for retroviral or lentiviral transduction, therefore, drives differentiation of T cells from a naïve towards a T_E_ phenotype [28]. In combination with ex-vivo culture protocols to expand transduced T cell numbers to those required for clinical application (~10^9^–10^11^), T cells are driven towards a more differentiated phenotype which is sub-optimal for systemic persistence.

With this in mind, the concept of isolating naïve or T_CM_ as a source for gene-modified T cells to reduce the extent of differentiation has been promoted with protocols described using paramagnetic beads to enrich CD62L^+^ (L-selectin) CD8^+^ T cells for transduction with a recombinant TCR [29]. CD62L expression characterises populations of T cells that possess lymphoid homing capability and is routinely used to identify naïve and effector memory T cells (T_EM_). However, CD62L expression on leukocytes is highly susceptible to freeze-thaw damage [30] and there is the suggestion that CD62L expression on CD4^+^ T cells is associated with a T_h_2 cytokine profile rather than preferred T_h_1 profile for cancer immunotherapy [31].

Similarly, expression of C-C motif chemokine receptor 7 (CCR7) identifies T cells that possess secondary lymphoid tissue homing capacity and T_CM_ characteristics (CCR7^+^) as compared to CCR7^−^ cells that possess effector function and the capability to migrate into inflamed tissue [26, 32]. We hypothesised that CCR7^+^ T cells may be optimal for adoptive T cell therapy. In this work, we report that CCR7^+^ T cells transduced with a TCR specific for a HLA-A*0201 restricted MART-1 epitope maintain many features associated with reduced differentiation and display enhanced engraftment in the NSG mouse model.

## Methods

### T cell subset isolation

Blood was collected from healthy donors or buffy coats (NHS Blood and Transplant Unit, Manchester, UK) and peripheral blood mononuclear cells (PBMC) isolated by Ficoll density centrifugation using Lymphocyte Separation Medium (PAA Laboratories GmbH, Austria). CD3^+^ T cells were enriched from PBMC by negative selection using a Pan T cell Isolation kit (Miltenyi Biotec, Bergisch-Gladbach, Germany). T cell subsets were labelled using monoclonal antibodies to enable positive selection of the relevant T cell populations. CCR7^+^ and CD62L^+^ T cells were isolated from enriched CD3+ T cell fractions using clones FR11-11E8 (Miltenyi Biotec, Germany) and DREG-56 (eBioscence, USA) respectively; both of which were conjugated to APC fluorophore. MACS separation based on APC (Miltenyi Biotec, Germany) was used to isolate CCR7^+^ or CD62L^+^ cells with negative fractions were collected as effluent. Weakly positive CCR7^+^ T cells were removed by passing cells from the CCR7^−^ fraction over a depletion column to allow residual CCR7^+^ cells to bind to the magnetic column. Where sorting was performed after cell culture, CD4^+^ T cells were depleted by positive selection (αCD4 antibody (RPA-T4-FITC, BD Bioscience, UK) and anti-FITC microbeads) using MACS depletion columns. Unlabelled CD8^+^ T cells were then labelled with αCCR7-APC or αCD62L-APC antibodies for before separation as described above.

### T cell activation

Non-selected, polyclonal T cell populations were activated by adding 30 ng/ml αCD3 (Orthoclone OKT3, OrthoBiotech, UK) and αCD28 (37407.111, R&D systems, UK) to 10^6^/mL PBMC in T cell media (RPMI 1640 media supplemented with 2 mM L-glutamine, 50 μM 2-mercaptoethanol, 10% foetal calf serum and 100 IU/mL Interluekin-2 (Chiron, The Netherlands). T cells which had been through a prior CD3^+^ enrichment process were activated, unless otherwise indicated, using human αCD3/αCD28 Dynabeads (Life Technologies, Norway) at concentration of 1 × 10^6^ T cells/ml in T cell media and a bead to T cell ratio of 1:1. MACSibeads (Miltenyi Biotec, Germany) were prepared by incubation of 10 μg/ml of each antibody (αCD2/αCD3/αCD28) with 10^8^ beads. Antibody-coated MACSibeads were then added to T cells (10^6^ T cells/mL) with a bead to T cell ratio of 1:2.

Irradiated PBMC feeder cells were generated by exposing PBMCs from three separate donors to 30 Gy irradiation before the addition of αCD3 and αCD28 antibodies, both at 0.5 μg/ml. Irradiated allogeneic feeder cells were added to cultures at a ratio of 2:1 T cells. K562 artificial antigen presenting cells (aAPC, a kind gift from Dr Carl June, University of Pennsylvania, USA) were exposed to 30 Gy irradiation and incubated with αCD3 and αCD28 antibodies (each at 0.5 μg/ml final concentration) prior to addition to T cell cultures at a ratio of 2:1 T cells. Activation using plate bound antibodies was carried out by coating αCD3 and αCD28 antibodies on non-tissue culture treated plates at a concentration of 1 μg/ml. Plates were washed and blocked with T cell media prior to the addition of T cells at 1 × 10^6^ cells/ml.

### T cell transduction

RetroDMF5 encoding the α and β chains of the MART-1 specific DMF5 TCR receptor was obtained from a stably transfected PG13 producer cell line. Viral supernatant was collected and passed through a 0.45 μm filter (Appleton Woods, UK) before immediate use or long-term storage at −80 °C. Transductions were carried out by spinfection using non-tissue culture 6 well plates coated in 10 μg/ml RetroNectin (Takara Bio, Japan). Briefly, activated T cells and virus were incubated overnight and the transduction procedure repeated the following day. Where T -cells were activated with magnetic beads, these were removed 4 h following the second spinfection. T cells were then seeded at a density of 5 × 10^5^/ml in T cell media with cytokines as indicated.

### Flow cytometry

Relative frequency of expression of specific markers were analysed using antibodies specific for αCD3, αCD8, αCD45RA, αCD27, αCD28, αCCR7 (BD Biosciences) and αCD62L (eBiosciences, USA). Where PBMCs analysed, Fc receptor block was used (Miltenyi Biotec, Germany). To detect DMF5 TCR expression, a MART-1/HLA*0201 antigen specific pentamer was used.

### Ex vivo functionality assays

RetroDMF5 transduced T cells were analysed simultaneously for both surface antigen expression and intracellular production of multiple cytokines via flow cytometry. Briefly, T cells were co-cultured with Mel-624 or Mel-888 cells at a ratio of 1:1 in the presence of 1 μl/ml GolgiPlug (BD Bioscience, UK) for 16 h at 37 °C. T cells were separated from from melanoma cell lines by a wash in 0.5 mM EDTA, then a second wash in FACS buffer. T cells were then stained for surface and intracellular antigens. T cell polyfunctionality was determined using SPICE v5.3 software (Simplified Presentation of Incredibly Complex Evaluations, distributed freely by National Institute of Allergy and Infectious Diseases, NIH).

The cytotoxic potential of DMF5 TCR transduced T cells was approximated by determining cell surface expression of CD107a following antigen specific T cell activation. Briefly, RetroDMF5 or mock transduced T cells were incubated with Mel-624 or Mel-888 at a ratio of 1:1 in T cell media with 1.2 μl/ml GolgiStop (BD Biosciences, UK). For analysis of CD107a expression, αCD107a-PE antibody (BD Biosciences) was added and cells incubated at 37 °C for 4 h, following which cells were washed, stained with αCD8-FITC (BD Biosciences) and analysed by flow cytometry. For analysis of IFNγ production, cells were incubated at 37 °C for 16 h prior to intracellular flow cytometry analysis.

### In vivo experiments

Human T cells were injected intravenously into NSG immunodeficient mice (Harlan, UK). Blood samples were collected by tail vein bleeds. All experiments were conducted under the auspices of the Animals (Scientific Procedures) Act 1986 and under U.K. Coordinating Committee for Cancer Research guidelines.

### Statistical analysis

Statistical analysis was performed using one-way or two way ANOVA as required with Bonferonni’s post-test. * *P* < 0.05, **P < 0.01, *** *P* < 0.001.

## Results

### CCR7 selection successfully enrich less differentiated T cells

**Fig. 1:**
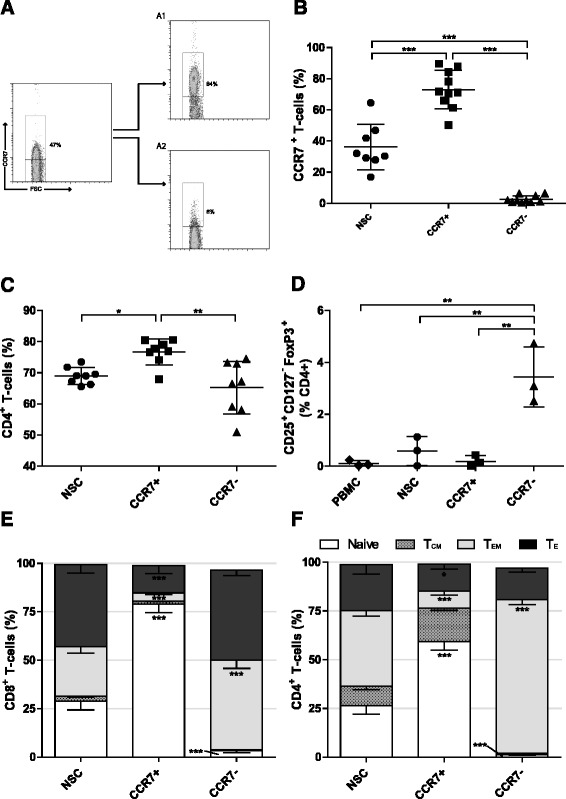
Early differentiated T cell selection using CCR7. **a** Representative plot following Isolation of early differentiated T cells using immuno-magnetic beads against CCR7. *A1* CCR7^+^ and *A2* CCR7^−^ fractions. The frequency of **b** CCR7^+^, **c** CD4^+^ and **d** CD25^+^ CD127^−^ FoxP3^+^ Tregs in each fraction following CCR7 selection as determined by flow cytometry. *NSC* Non sorted control T cells. *Error bars* ± SD, statistical tests one way ANOVAs with Bonferroni’s post-test. **e** and **f** The phenotype of CD8^+^ and CD4^+^ T cell populations respectively following CCR7 selection; Naïve (CCR7^+^ CD45RA+), Central memory (CCR7^+^ CD45RA^−^), Effector memory (CCR7^−^ CD45RA^−^), Effector (CCR7^−^ CD45RA^+^). *Error bars* show ± SEM. For each phenotype; significant difference from NSC T cells is shown. * *P* < 0.05, **P < 0.01, *** *P* < 0.001

The chemokine receptor CCR7 is expressed on naïve, stem cell memory (T_SCM_) and central memory (T_CM_) T cells [27]. We developed an antibody-based paramagnetic bead isolation approach to enrich CCR7^+^ T cells from PBMCs to enrich for these early-differentiated T cell subsets. The process involves enriching CD3^+^ T cells from PBMCs by paramagnetic bead-based negative selection then a further two-stage bead based isolation strategy employing an initial staining of T cells with an APC-conjugated anti-CCR7 antibody, followed by incubation with anti-APC paramagnetic microbeads and magnetic separation. Flow cytometry of positive and negative fractions of cells confirms the ability of this process to enrich for T cells expressing CCR7 (Fig. 1a). Despite the wide variability in the relative proportion of CCR7^+^ cells within the CD3^+^ T cell population of healthy donors (mean: 36.2% SD ± 14.6%, range: 16.9–64.5%), CCR7^+^ T cells were routinely enriched about 2 fold to to 73.1% (SD ± 12.3%) while the negative fraction was depleted about 14 fold to a level of 2.5% (SD ± 2.3%) CCR7^+^ T cells (Fig. 1b).

The frequency of CD4^+^ T cells in CCR7^+^ selected cell populations was 76.7% (SD ± 4.13%), significantly higher than 69.0% (SD ± 2.68%) in non-selected T cells (NSCs; *P* < 0.05) and 65.2% (SD ± 8.48%) present in CCR7^−^ cell fractions (*P* < 0.01; Fig. 1c). Rises in CD4^+^ T cell frequency were concomitant with a reduction in CD8^+^ T cell frequency (Additional file 1A). CCR7 expression has been identified in certain subsets of regulatory T cells (Tregs) [33], which, if enriched, could inhibit effector functions of adoptively transferred T cells in vivo. However, there was no increase in the relative frequency of CD25^+^ CD127^−^ FoxP3^+^ Tregs within the CCR7^+^ T cell population. Unexpectedly, there was a 4.2 fold increase in Tregs in the CCR7^−^ population as compared to NSCs (*P* < 0.01; Fig. 1d).

Based upon reported determinants of T cell differentiation status [27, 34], CCR7 selection enriched naïve (CCR7^+^ CD45RA^+^) T cells in both CD4^+^ and CD8^+^ compartments (2.2 and 2.7 fold respectively) and caused a reduction in both effector memory (T_EM_, CCR7^−^ CD45RA^−^) (4.4 and 6.1 fold respectively) and effector (T_E_, T_EMRA_/effector, CCR7^−^ CD45RA^+^) T cells (1.7 and 3.0 fold respectively) when compared to NSCs. Conversely, a reduction in the proportion of naïve and an increase in T_EM_ in CD4^+^ and CD8^+^ subsets within the CCR7^−^ population was observed (Fig. 1e and f). A trend of increased relative expression of both CD27 and CD62L^hi^ in CCR7^+^ populations was not statistically significant relative to NSCs. Simultaneous decreases within CCR7^−^ populations, however, were indeed. Relative proportions of CD28 expressing cells were unchanged regardless of CCR7 expression (Additional file 1B and C). Together, these data show that selection based on CCR7 enriches for T cells bearing an early-differentiated marker phenotype without co-enriching Tregs.

### The context of mitogenic stimulation influences the proliferation kinetics and phenotypic profile of non-selected and CCR7^+^ T cells

**Fig. 2:**
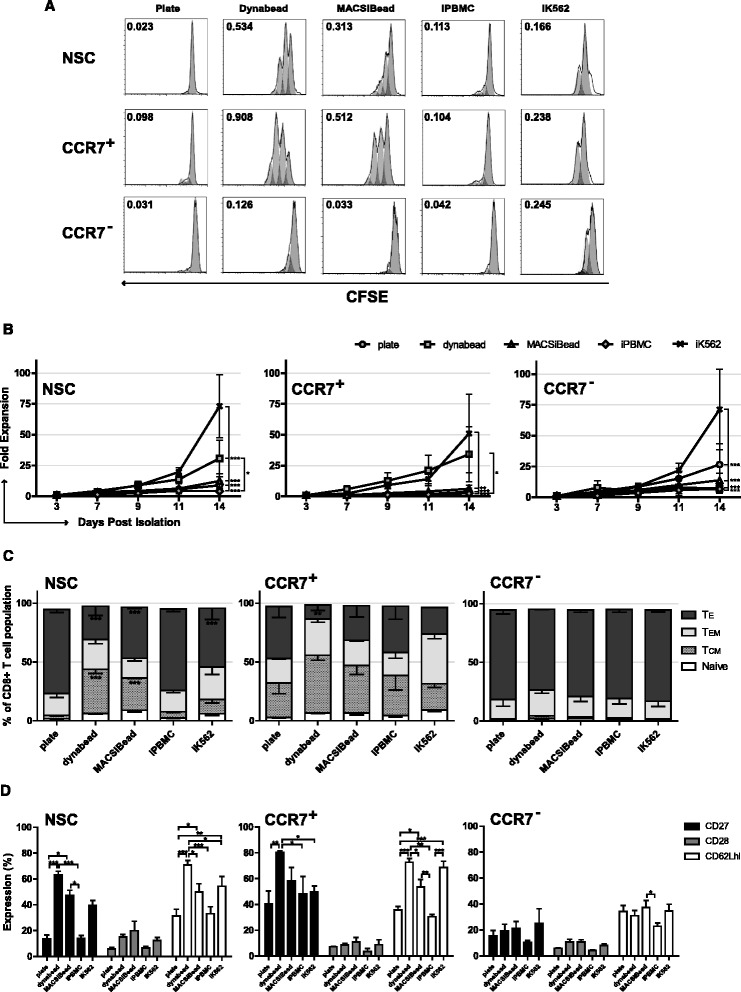
Proliferation kinetics and phenotype following various mitogenic stimuli. **a** Representative flow cytometry data 72 h following CFSE staining and activation of T cells with mitogenic agents after selection for expression of CCR7. **b** Long term proliferation of T cells following selection for expression of CCR7, mitogenic activation, transduction with RetroDMF5 and culture in IL-2. Expansion of NSC, CCR7^+^ and CCR7^−^ T cell populations was assessed after 14 days through enumeration of live cells. **c** The phenotype CD8^+^ T cell populations following CCR7 selection and **d** Analysis of additional markers of early differentiation CD27, CD28 and CD62Lhi. *Error bars* show ± SEM. For phenotypes, significant difference from plate bound antibody is shown

Optimal retroviral transduction of human T cells occurs 48 h after mitogenic stimulation [35]. To analyse whether the context of anti-CD3ε and anti-CD28 (αCD3/αCD28) mitogenic antibodies had a differential impact upon T cell subset proliferation, NSC, CCR7^+^ and CCR7^−^ cell populations were stimulated with antibodies in different contexts: plate bound αCD3/αCD28, microbead bound αCD3/αCD28 (dynabeads), cell sized microbead bound αCD2/αCD3/αCD28 (MACSiBeads) and presentation of αCD3/αCD28 by irradiated PBMCs (iPBMCs) and irradiated K562 aAPC (iK562). A clear difference in the ability of these divergent platforms to induce the short-term proliferation of NSC, CCR7^+^ or CCR7^−^ CD8^+^ T cell fractions was found. Both dynabeads and MACSiBeads induced a rapid burst of proliferation in CCR7^+^ and NSC T cells above that observed with plate-bound or cell based antibody presentation (Fig. 2a). CCR7^−^ cells displayed much lower proliferative capacity, characteristic of a more differentiated phenotype, although iK562 cells induced the highest proliferation of CCR7^−^ cells over 72 h (Fig. 2a). Despite these differences in short-term proliferation, no significant difference in transduction efficiency with dynabeads or MACsiBeads platforms was observed (Additional file 2A).

Following transduction and IL-2 driven ex vivo culture for 14 days, iK562 cells induced significantly greater expansion of NSCs than all other antibody activation methods whilst dynabeads induced significantly greater proliferation than iPBMCs (Fig. 2b). Dynabead activation was also associated with the lowest relative proportion of T_E_ and also yielded the highest proportions of T_CM_ within the NSC population (Fig. 2c). Moreover, there were significantly higher numbers of CD27^+^ cells upon activation of NSCs with dynabeads and MACSiBeads, compared with plate bound antibodies or iPBMCs (Fig. 2d). Dynabeads also maintained the highest frequency of CD62L^hi^ T cells as compared to all other platforms (Fig. 2d).

In the case of CCR7^+^ selected cells, iK562 cells induced significantly greater expansion than MACSiBeads, iPBMCs and plate bound antibodies (*P* < 0.001) whilst dynabeads induced significantly greater proliferation than iPBMCs. Importantly, there was no significant difference in the expansion of CCR7^+^ selected cells over 14 days in the presence of iK562 cells and dynabeads (Fig. 2b). The relative proportion of T_CM_ was greater than all equivalent conditions compared to NSCs. Moreover, the frequency of T_E_ cells was significantly decreased in dynabead, activated cells compared to CCR7^+^ cells activated with plate bound antibody (3.78 fold) or iPBMCs (3.37 fold) and the proportion of T_CM_ significantly increased compared to iK562 activation (2.21 fold; Fig. 2c).

In CCR7^−^ selected T cells, iK562 cells induced significantly greater expansion than all other platforms (Fig. 2b). Within the CCR7^−^ derived populations, there was no significant effect of mitogenic stimulation on differentiation, with all of the populations consisting of equivalent numbers of effector memory and effector cells (Fig. 2c) with the only significant difference in surface marker expression being a small but significant increase in CD62L^hi+^ cells between MACSiBead and iPBMC activated cells (*P* < 0.05).

To summarise, short term proliferation was maximal in cells activated with dynabeads and MACSiBeads, with maximal 14-day expansion of CCR7^+^ T cells achieved with dynabeads and iK562 cell αCD3/αCD28 antibody activation. There was little variation in T_EM_ populations regardless of CCR7 selection or mitogen used. This could be an artefact derived from T cell activation itself, retrovirus transduction or cell culture conditions; alternatively, it may be reflective of normal T cell subsets. However, these phenotype data show that activation of unselected CD8^+^ T cells or CD8^+^ CCR7^+^ T cells with dynabeads is superior to other mitogenic platforms assessed at expanding T cells whilst retaining expression of markers associated with early-differentiation status, in particular yielding higher proportions of T_CM_ and limiting differentiation to T_E_.

### CCR7^+^ selection yields younger T cells than CD62L^+^ selection

Having validated the ability of CCR7 selection to enrich for minimally differentiated T cells and identifying dynabeads as an ideal mitogen to retain maximal levels of T_CM_ cells upon cell expansion, we compared this to an analogous method being developed to enrich for early-differentiated T cells for, selection based on CD62L expression [36–38].

**Fig. 3:**
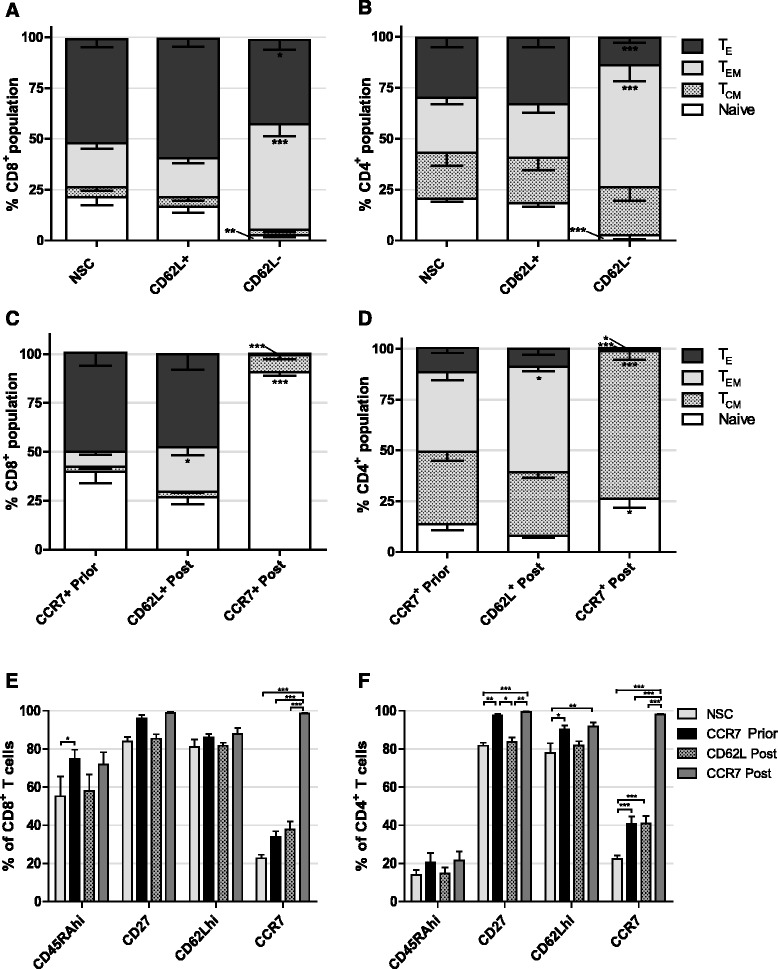
Effect of CD62L selection and timing of CCR7 selection on T cell phenotype. **a** CD8^+^ and **b** CD4^+^ T cell phenotypes following CD62L selection. Significant difference from NSC T cells is shown. **c** CD8^+^ and **d** CD4^+^ T cell phenotypes following CCR7 selection prior to, or CD62L or CCR7 selection post 14 days expansion ex vivo. Significant difference from CCR7 prior to expansion is shown. **e** CD8^+^ and **f** CD4^+^ T cell expression of additional early differentiation markers following selection prior to, or post expansion ex vivo. *Error bars* show ± SEM

Firstly we investigated whether selection based on CD62L expression could enrich for T cells of early-differentiation status. As with CCR7 selection, APC conjugated αCD62L and αAPC beads were used to separate cells. In contrast to work elsewhere, upon phenotypic analysis of separated cells there was no significant increase in proportion of Naïve or T_CM_, nor decrease in T_E_ following CD62L selection in either CD8^+^ or CD4^+^ T cells compared to NSCs (Fig. 3a and b). Conversely, CD62L^−^ cells contained enlarged proportions of T_EM_ in both CD8^+^ and CD4^+^ subpopulations (*P* < 0.001 in each case) and a concomitant decrease in both naïve (*P* < 0.01 and *P* < 0.001 respectively) and T_E_ (*P* < 0.05 and *P* < 0.001 respectively) (Fig. 3a and b).

Selecting immature cells at the start of cell culture offers the advantage that cytokines and growth factors and nutrients are only consumed by cells of desired phenotype and unwanted cells are discarded immediately. However, despite enriching for less-differentiated cells prior to cell culture we consistently observed significant differentiation upon T cell expansion. We therefore investigated whether selection after expansion of NSCs was superior for acquiring minimally differentiated T cells compared to CCR7 selection prior to expansion.

Following activation with dynabeads, retroviral transduction and a 14 day period of cell culture, T cells were sorted for either CD62L or CCR7 expression. Results showed that, in comparison to CCR7 selection prior to cell culture, CD62L selection at the end of cell culture significantly increased the proportion of CD8^+^ (3.0 fold *P* < 0.05) and CD4^+^ (1.32 fold *P* < 0.05) T_EM_ populations, however in both cases there was a trend of reduced naïve cells. CCR7 selection post expansion led to a drastic 2.3 fold increase (*P* < 0.001) in the population of CD8^+^ naive T cells up to 93.8% and simultaneous 67.7 fold decrease in T_E_ proportion (*P* < 0.001) (Fig. 3c). In CD4^+^ cells, CCR7 selection did lead to a 1.9 fold increase in naïve subsets (*P* < 0.05), but to a lower total of 26.3% whilst T_CM_ were increased in numbers 2.0 fold (*P* < 0.001), complementary decreases in T_EM_ and T_E_ were also observed (Fig. 3d). Interestingly, CD62L nor CCR7 selection following expansion in cell culture resulted in increased proportion of the additional markers of early-differentiation CD27 or CD62Lhi in CD4^+^ or CD8^+^ compartments compared to CCR7 selection prior to expansion (Fig. 3e and f).

### CCR7^+^ selected cells display reduced exhaustion and senescence

**Fig. 4:**
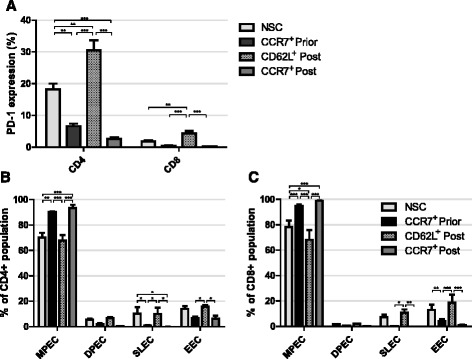
Exhaustion and senescence following T cell selection. **a** PD1 expression of CD8^+^ and CD4^+^ T cells following selection prior to, or post 14 days expansion ex vivo. Statistical analysis was performed using two separate one-way ANOVAS. **b** CD8^+^ and **c** CD4^+^ T cell subpopulations of memory precursor effector cells (MPECs) KLRG1^−^ CD127^+^, double positive effector cells (DPECs) KLRG1^+^ CD127^+^, short lived effector cells (SLECs) KLRG1^+^ CD127^−^ or early effector cells (EECs) KLRG1^−^CD127^−^ following selection prior to, or post 14 days expansion ex vivo. *Error bars* show ± SEM

We assessed the expression of PD1, a marker of exhaustion, after expansion of cells in vitro (Fig. 4a). Our first observation was that about 10 fold more CD4^+^ cells than CD8^+^ cells expressed PD1; 18.2% compared to 1.8% respectively. Secondly, CD62L selection after 14 days expansion resulted in significantly higher levels of PD-1 expression compared to CCR7 selection in both CD8^+^ and CD4^+^ subsets prior to (8.8 and 4.6 fold respectively), or post expansion (25.4 and 11.7 fold respectively) (*P* < 0.001 in all cases). Interestingly, CD62L selection led to more cells showing exhaustion than NSCs in both CD8^+^ (2.4 fold) and CD4^+^ (1.7 fold) subsets (*P* < 0.01 in both cases). CCR7 selection either prior to, or post expansion yielded a significant decrease in CD4^+^ cells expressing PD-1 compared to NSCs from 18.2 to 6.62% (*P* < 0.01) and 2.61% (*P* < 0.001) respectively.

In addition to PD1 as a marker of exhaustion, we analysed the expression of killer cell lectin-like receptor 1 (KLRG1) and CD127, a subunit of the IL-7 receptor; predicators of the replicative potential of T cells in vivo [39]. Based on expression of these markers we identified memory precursor effector cells (MPECs, KLRG1^−^ CD127^+^), double positive effector cells (DPECs, KLRG1^+^ CD127^+^), short lived effector cells (SLECs, KLRG1^+^ CD127^−^) or early effector cells (EECs, KLRG1^−^CD127^−^). MPECs have large replicative potential and make up T_EM_ and T_CM_ whilst SLECs and EECs are lost over time though apoptosis. We observed about a 20% increase in numbers of MPECs upon CCR7 selection either prior to, or post T cell expansion compared to NSCs in CD4^+^ (*P* < 0.01 and *P* < 0.001 respectively) and CD8^+^ cells (*P* < 0.001 in both cases) (Fig. 4b and c). Notably, there was no change in the yield of MPECs whether cells were selected for CCR7 before or after expansion. Conversely, compared to NSCs, CD4^+^ SLECs were significantly reduced by CCR7 selection (*P* < 0.05) and CD8^+^ EECs were decreased by CCR7 selection prior to expansion (*P* < 0.01). Consistent with observed increased PD-1 expression, CD62L selection did not increase the yield of MPECs nor decrease the yield of SLECs or EECs compared to NSCs in both CD4^+^ and CD8^+^ populations (Fig. 4b and c).

### Functionality of CCR7^+^ selected cells

CCR7 selection yields T cells of early-differentiation status with potential for long-term persistence in vivo. Efficacy of CAR T cell therapy, however, is also dependent upon effector function of CAR T cells. We therefore assessed the cytokine production and cytotoxicity of selected T cells.

**Fig. 5:**
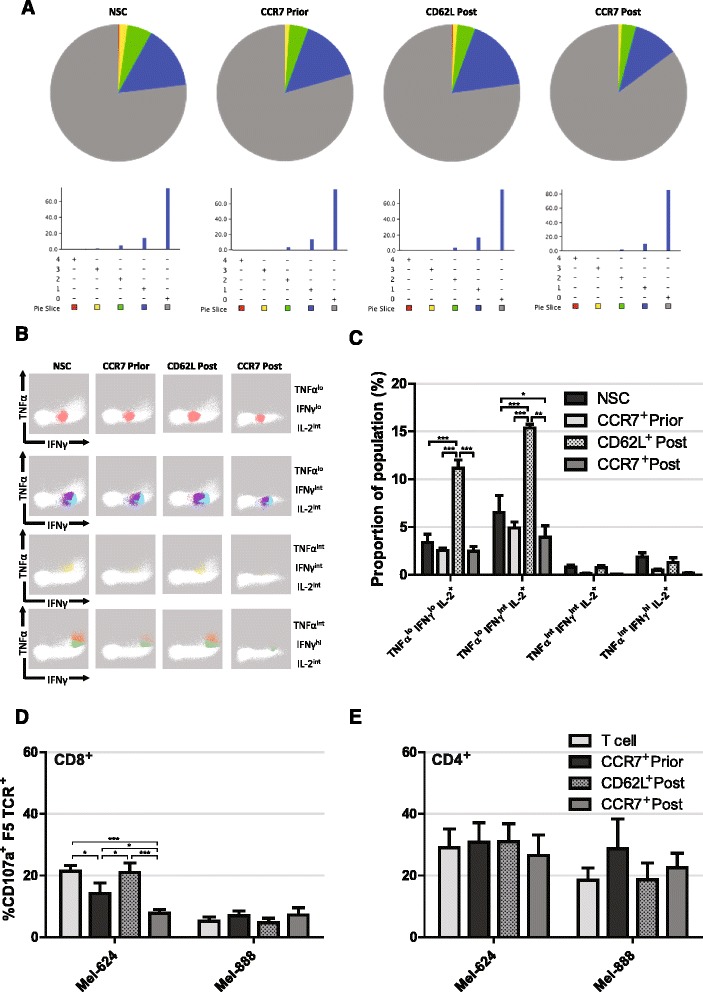
Functionality of selected T cells. **a** Representative pie charts of T cell proportions secreting 0 – *grey*, 1 – *blue*, 2 – *green*, 3 – *yellow* or 4 – *red* cytokines of a panel consisting of IFNγ, IL-2, IL-10, IL-17A and TNFα upon 16 h incubation with Mel-624 cells. **b** Visual representation and **c** quantification of IL-2 secreting cell subsets following CCR7 selection prior to, or CD62L or CCR7 selection post expansion ex vivo and incubation with target Mel-624 cells. Manual gating was utilised to identify CD8^+^ T cells and data from 4 biological repeats were pooled before FLOCK analysis. **d** CD8^+^ and **e** CD4^+^ T cell CD107a expression following selection prior to, or post expansion and incubation with target Mel-624 cells. *Error bars* show ± SEM

As the DMF5 TCR is specific for an epitope of MART-1 when presented on HLA-A2, cytokine production of selected T cells upon encountering target antigen was analysed by measuring production of IFNγ, IL-2, IL-10, IL-17A and TNFα upon 16 h incubations with the MART-1^+^, HLA-A2^+^ melanoma cell line, Mel-624. Equivalent numbers of RetroDMF5 transduced T cells that were either non-sorted, CCR7 selected prior to in vitro expansion, CCR7 selected post expansion or CD62L selected post expansion were incubated with target Mel-624 cells. Using SPICE application software [40], the multifunctionality of T cells was characterised by the number of measured cytokines produced. We observed little variation in multifunctionality of NSC, CCR7 or CD62L selected, RetroDMF5 transduced T cells. Although CCR7 selection post expansion seemed to yield a slightly increased proportion of cells secreting no cytokines after co-culture with target Mel-624 cells (Fig. 5a).

It has been shown that IL-2 production is required for the generation of CD8^+^ memory T cells with the ability to mount secondary responses [41]. After incubation of DMF5 transduced T cells with target Mel264 cells we identified IL-2 producing CD8^+^ T cells and further analysed them based on their production of TNFα, IFNγ, IL-10 and IL-17A via flow cytometry, however, neither IL-10 nor IL-17A were co-produced with IL-2. FLOCK (FLOw Cytometry without K) analysis (http://www.immport.org/immport-open/public/home/flowAnalysis) provides an algorithm to functionally characterise cells based on several surface markers without the bias of manual gating. FLOCK was used to identify functional subpopulations of IL-2 producing CD8^+^ T cells based on high, intermediate, or low levels of TNFα and IFNγ production. Results showed a clear association of IL-2 production with TNFα^lo^, IFNγ^lo^ and TNFα^lo^, IFNγ^int^ producing T cells upon incubation with target melanoma cells (Fig. 5b and c). In addition, CD62L selection post expansion gave rise to over a 3 fold increase in numbers of TNFα^lo^, IFNγ^lo^, IL-2 producing cells than NSC or CCR7 selected cells both pre and post expansion (*P* < 0.001 in all cases) and over a two fold increase in numbers of TNFα^lo^, IFNγ^int^, IL-2 producing cells than NSC or CCR7 selected cells both pre and post expansion (*P* < 0.001, *P* < 0.001 and *P* < 0.01 respectively). Of note, CCR7 selection post expansion led to a 40% reduction in the proportion TNFα^lo^, IFNγ^int^, IL-2 producing cells compared to NSCs (*P* < 0.05).

As well as cytokine release, direct cytotoxicity of T cells was assessed by measuring levels of CD107a, a marker of degranulation and therefore direct cytotoxicity, upon incubation of T cells with target melanoma cells. In order to confirm MART1-HLA-A2 specific activation of RetroDMF5 transduced T cells, Mel-888 cells that express MART-1 and HLA-A1 were used as a control to quantify any non-specific activation. Firstly, it was apparent that there was little non-specific activation of CD8^+^ T cells by Mel-888 cells (Fig. 5d). Secondly, we observed a high level non-specific activation of CD4^+^ T cells (Fig. 5e); this could be an experimental artefact or perhaps reflective of recent work suggesting CD4^+^ T cell degranulation is less tightly regulated than that of CD8^+^ counterparts [42].

CCR7 selection of T cells post expansion led to reduced cytotoxicity compared to NSC (3.1 fold, *P* < 0.001), CCR7 selection prior to expansion (1.6 fold, *P* < 0.05) and CD62L post expansion (3.6 fold, *P* < 0.001) to levels similar to background (Fig. 5d). CCR7 selection prior to expansion also led to a reduction in cytotoxicity compared to NSCs (1.9 fold, *P* < 0.05) and CD62L selection post expansion (2.2 fold, *P* < 0.05) (Fig. 5d).

Together, these data suggest CCR7 cells have lower short-term direct cytotoxicity to target tumour cells, which we and others propose is indicative of an early-differentiated phenotype and increased capacity of long term engraftment in vivo, a trait required for tumour eradication.

### CCR7 selected human T cells exhibit enhanced engraftment in vivo

Given that data suggests CCR7 selected T cells display multiple characteristic characteristics of early-differentiation status we tested whether this led to enhanced engraftment and reduction in tumour growth in the severely immunodeficient NSG (NOD/SCID IL-2Rγ^-/-^) mouse strain.

The NSG mouse model required to study the long term effects of human T cells in mice is hampered by the transferred T cells reacting to mouse antigens. The immune reaction increases in intensity over time until at around 50 days post T cell transfer when mice must be sacrificed due to xenogenic graft versus host disease [43–46]. This effect limits the time scale of tumour eradication studies for CCR7 sorted T cells. Despite this we analysed whether CCR7+ had more anti-tumour potency than CCR7- magnetically sorted DMF5 TCR transduced T cells over the course of 3 weeks in NSG subcutaneously inoculated with Mel624 tumours. Although there was a trend of a 2 fold reduction in tumour volume after 3 weeks, results were not statistically significant by two-way ANOVA (Additional file 2B).

To study the engraftment of adoptively transferred T cells. Following transduction of with the RetroDMF5 TCR a dose of 2 × 10^7^ non-sorted T cells or varying doses of equivalent CCR7 selected cells were administered to mice via intravenous (IV) injections. Blood samples collected by tail bleeds were analysed by flow cytometry both 1 and 3 weeks following IV injections.

**Fig. 6:**
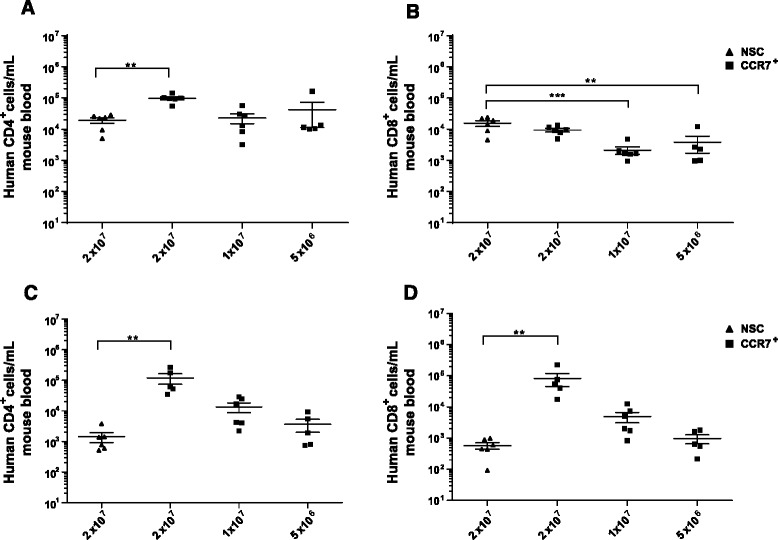
Persistence of selected T cells in in vivo models. NSG mice were injected intravenously with CCR7 sorted or Non Sorted T cells and analysed for circulating levels of adoptively transferred and T cells on days 7 **a** and **b** and 21 **c** and **d**; CD4^+^ and CD8^+^ respectively. *Error bars* show ± SEM

Upon analysis of the numbers of adoptively transferred human CD4^+^ T cells in the circulation after 1 week the numbers of human CD4^+^ T cells were 5.10 fold higher (*P* < 0.01) in mice that received 2 × 10^7^ CCR7 selected cells than mice that received an equivalent dose of NSCs. There was also no significant difference in numbers of circulating human CD4^+^ numbers in mice receiving 2 × 10^7^ NSCs and 2 fold or 4 fold lower initial doses of CCR7 selected cells (Fig. 6a). After 3 weeks it was observed that CCR7 selection led to an 83.6 fold increase (*P* < 0.01) in circulating CD4^+^ cells compared to NSCs at an equivalent dose of 2 × 10^7^ cells and a non-statistically significant rise in mice receiving both 2 fold and 4 fold lower doses of CCR7 selected cells (Fig. 6c).

Analysis of circulating human CD8^+^ T cells 1 week after adoptive transfer showed there was no significant increase in numbers in mice receiving 2 × 10^7^ NSCs compared those receiving an equivalent dose of CCR7 selected cells. In fact there was a significant decrease in numbers of circulating CD8^+^ T cell numbers in mice receiving or 1 × 10^7^ (6.81 fold, *P* < 0.001) or 5 × 10^6^ (3.78 fold, *P* < 0.01) CCR7 selected cells compared to those receiving 2 × 10^7^ NSCs. This reflects reduced input doses and perhaps an intrinsically different efficiency of engraftment or proliferation of RetroDMF5 transduced CD8^+^ T cells compared to CD4^+^ T cells. After 3 weeks, however, we observed a 137 fold increase (*P* < 0.01) in circulating human CD8^+^ T cell numbers in mice that received 2 × 10^7^ CCR7 selected cells than those that received an equivalent dose of NSCs. Similar to the CD4^+^ circulating cells, a non-statistically significant increase in numbers were also observed in mice that received both 2 and 4 fold lower doses of CCR7 selected cells than those that received 2 × 10^7^ NSCs.

## Discussion

We demonstrate here that a bead based selection of CCR7 expressing cells efficiently selects for T cells of early differentiation status, significantly enhancing the proportion of naïve cells whilst decreasing the prevalence of T_EM_ and T_E_ cells. This method has a slight bias for CD4^+^ T cells, probably reflecting the proportion of circulating cells in vivo. Despite a CD4^+^ bias, there was no increase in CD4^+^ CD25^+^ CD27^−^ FoxP3^+^ Tregs, which may have a negative effect on anti-cancer potency of adoptively transferred T cells.

Upon comparison of a range of mitogenic agents operating through αCD3 and αCD28 antibodies, we identified dynabeads as an ideal agent for induction of both short and long term proliferation of CCR7 sorted T cells whilst inducing the least differentiation over a 2 week period. This was evidenced by increased levels of CD27 and CD62Lhi expressing cells and reduced T_E_ numbers. These observations are supported by data published elsewhere but encompass a more complete panel of available mitogenic agents, specifically with relation to gene modified T cells enriched for early-differentiation status [47, 48].

In addition to CCR7, we attempted to enrich for cells of early-differentiation status by utilising CD62Lhi, another marker of early-differentiation. This did not prove efficacious, perhaps due the presence of lower levels of CD62L being present on more differentiated cells.

We assessed functional characteristics of T cells postulated to correlate with in vivo persistence. CCR7 selection either prior to, or post T cell expansion led to cells with reduced PD1 expression, indicating decreased levels of exhaustion, as well as increased markers associated with high proliferative capacity. In combination with phenotypic markers of early-differentiation, this indicates that CCR7 selected cells have increased capacity for persistence patients in vivo.

Upon incubation of CCR7 sorted, gene modified T cells with target melanoma cells, multifunctionality was largely unchanged compared to non-sorted T cells. Analysis of IL-2 producing CD8^+^ T cells following incubation with target Mel624 cells showed an increase in TNFα^lo^, IFNγ^lo^ and TNFα^lo^, IFNγ^int^ IL-2 secreting populations in CD62L sorted cells. In all cases the number of TNFα and IFNγ producing IL-2 secreting CD8 T cells was lower in CCR7 sorted groups. As these have synergistic effects on T cell proliferation this may be reflective of a less effector and early differentiated phenotype, concomitant with other data presented here [49].

Importantly, CCR7 selection either prior to, or post expansion led to a decrease in cytotoxicity of TCR-transduced T cells upon incubation with target melanoma cells. This signifies early differentiation status and, paradoxically, implies potential for higher anti-cancer efficacy in vivo, owing to increased capacity for long term persistence.

Analysis of the persistence of human gene modified T cells in mouse models are severely hampered by x-GvHD therefore it is not possible to analyse human T cells in mice in the long term [44]. However, it is possible to analyse engraftment levels of human T cells in severely immunodefecient NSG mice before such xenoreactivity develops. Using this model we showed a drastic increase in the numbers of adoptively transferred cells in mice receiving CCR7 sorted cells over standard, non-sorted cells. Upon comparison with CD62L sorted T cells, which were of a more differentiated phenotype in vitro, engraftment levels were similar after 3 weeks. We hypothesised that over an extended period CCR7 sorted cells would outperform CD62L sorted cells, however, this was not evaluable in the NSG mouse model due to the necessity to sacrifice mice after 4–6 weeks as a result of x-GvHD.

We suggest that in order to validate the effects of CCR7 selection on persistence of human cells in vivo, clinical investigation must take place.

## Conclusion

Sufficient levels of persistence of adoptively transferred T cells are key for successful clinical outcomes. There is also growing evidence that the proliferative capacity of T cells is a principle determinant for efficient engraftment and tumour clearance. It is, therefore, paramount, to determine optimal conditions for the production of T cells with maximum proliferative potential on a clinical scale. This study provides evidence that bead based selection of CCR7^+^ T cells prior to adoptive cell therapy may provide a means to enhance engraftment levels and may therefore improve clinical success in multiple applications.

## Additional files

Additional file 1: CD8 cell frequency and additional markers of differentiation. A) CD8+ frequency following CCR7 selection of T-cells following isolation. B) CD8^+^ and C) CD4^+^ T-cell expression of early differentiation markers following CCR7 selection upon isolation. Error bars show SEM. Statistical analysis was performed using one-way or two-way ANOVA with Bonferroni’s post-tests. (TIF 17637 kb)
Additional file 2: Transduction levels and inhibition of Mel624 tumour growth in NSG mice. A) RetroDMF5 transduction efficiency of CCR7 selected T-cells following activation with various mitogenic agents. Error bars show SEM. Statistical analysis was performed using one-way ANOVA with Bonferroni’s post-test. B) T cells were transduced with the DMF5 TCR and magnetically sorted into CCR7+ and CCR7- fractions. These cells were transferred to NSG mice with palpable, subcutaneous Mel624 tumours. Tumour growth was assessed over the course of 3 weeks. Error bars show + SEM. Statistical analysis was performed using two-way ANOVA. (TIF 15008 kb)

## Abbreviations

ATCT: Adoptive T cell immunotherapy
CAR: Chimeric antigen receptor
CCR7: C-C motif chemokine receptor 7
CD62L^+^: L-selectin+
NSCs: Non-selected T cells
PBMC: Peripheral blood mononuclear cells
T_CM_: Central memory T cell
TCR: T cell receptor
T_E_: Effector T cell
T_EM_: Effector memory T cell
Treg: Regulatory T cell
T_SCM_: Stem cell memory
