# Supplementary figures and images for: CCR7+ selected gene-modified T cells maintain a central memory phenotype and display enhanced persistence in peripheral blood in vivo
Source: J Immunother Cancer. 2017 Feb 21;5:14. doi: 10.1186/s40425-017-0216-7 (PMC5319186; doi:10.1186/s40425-017-0216-7)

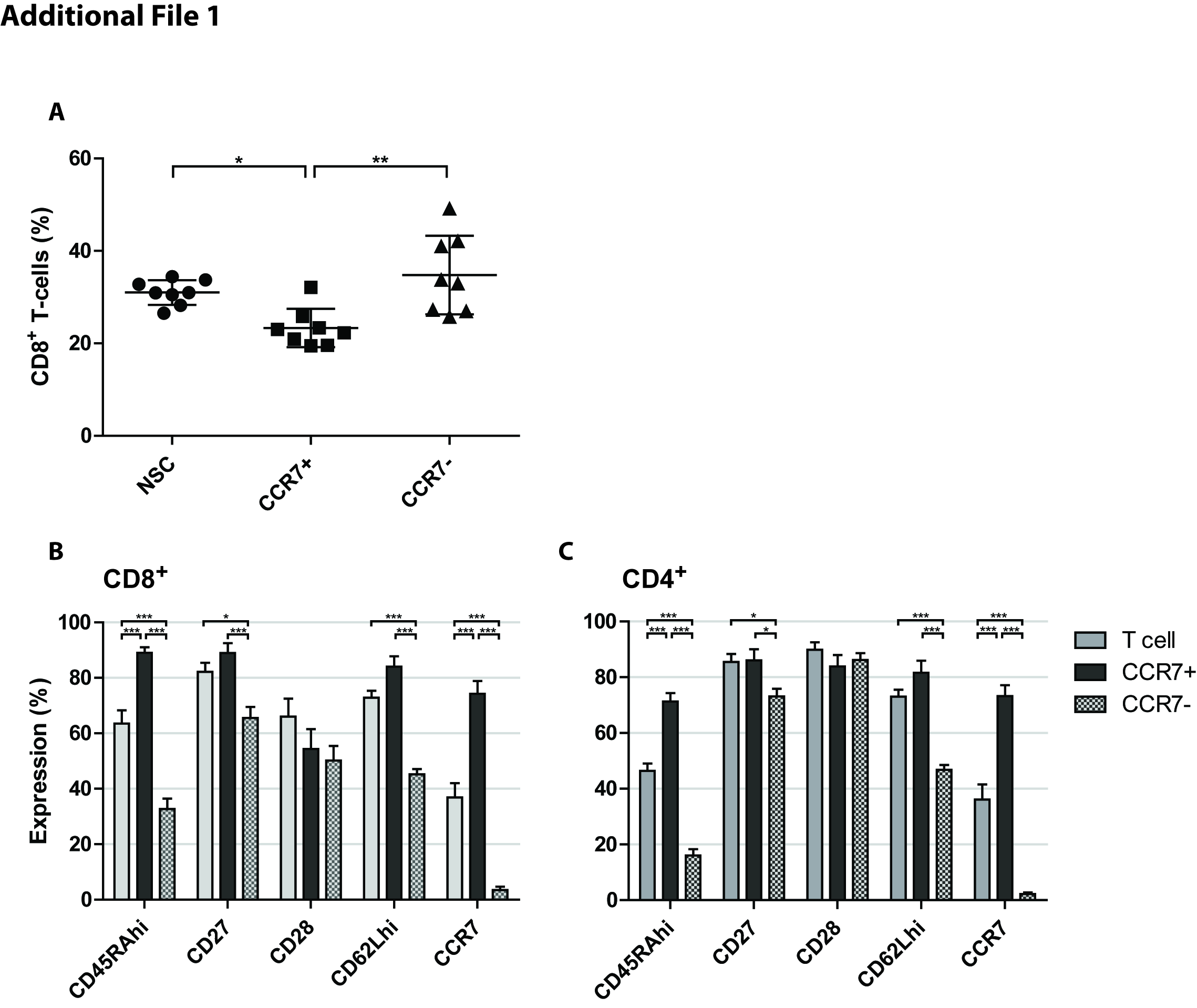

Supplement: Additional file 1: — CD8 cell frequency and additional markers of differentiation. A) CD8+ frequency following CCR7 selection of T-cells following isolation. B) CD8+ and C) CD4+ T-cell expression of early differentiation markers following CCR7 selection upon isolation. Error bars show SEM. Statistical analysis was performed using one-way or two-way ANOVA with Bonferroni’s post-tests. (TIF 17637 kb) [file 40425_2017_216_MOESM1_ESM.tif]

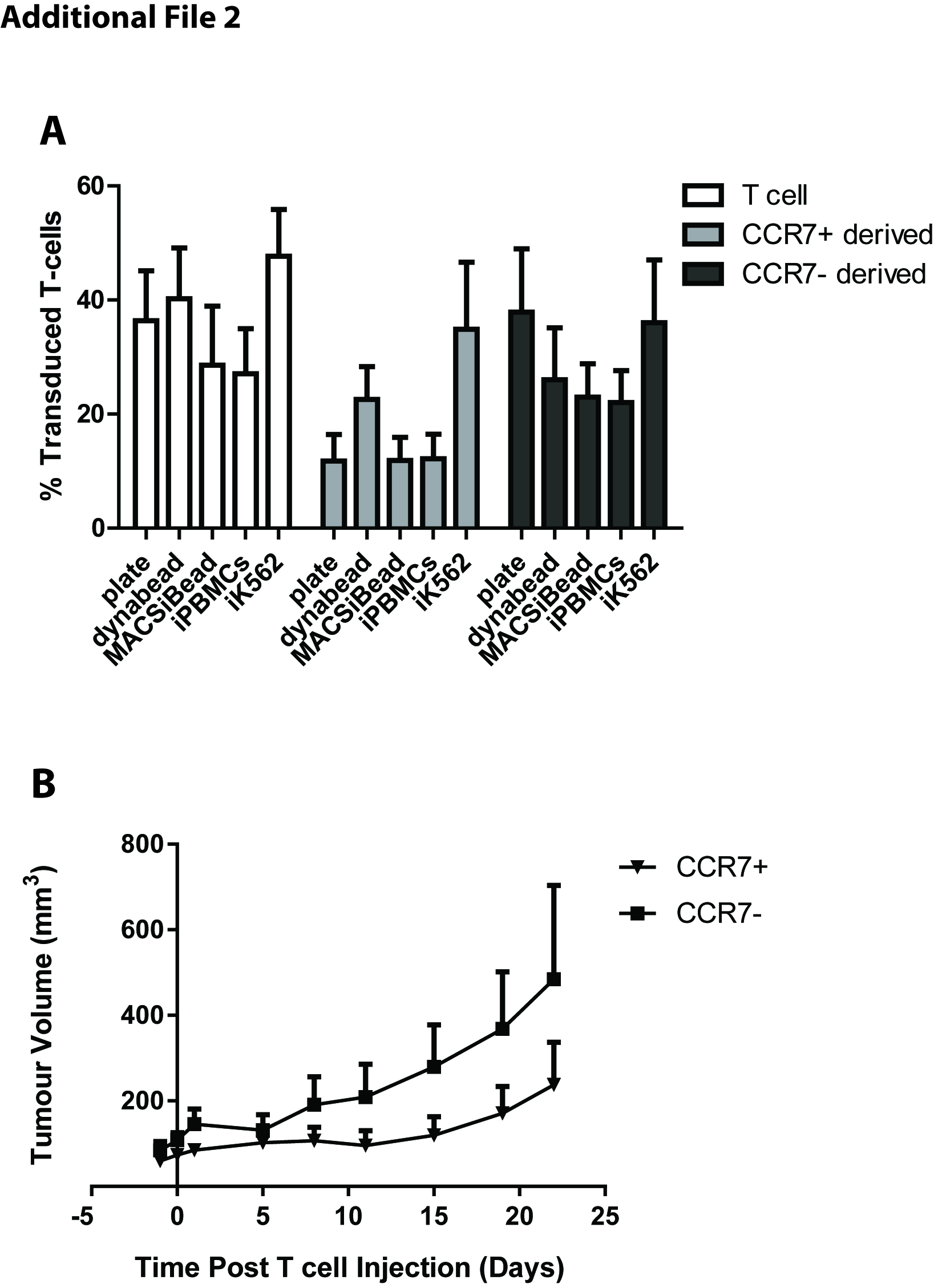

Supplement: Additional file 2: — Transduction levels and inhibition of Mel624 tumour growth in NSG mice. A) RetroDMF5 transduction efficiency of CCR7 selected T-cells following activation with various mitogenic agents. Error bars show SEM. Statistical analysis was performed using one-way ANOVA with Bonferroni’s post-test. B) T cells were transduced with the DMF5 TCR and magnetically sorted into CCR7+ and CCR7- fractions. These cells were transferred to NSG mice with palpable, subcutaneous Mel624 tumours. Tumour growth was assessed over the course of 3 weeks. Error bars show + SEM. Statistical analysis was performed using two-way ANOVA. (TIF 15008 kb) [file 40425_2017_216_MOESM2_ESM.tif]
